# Comparison of stereotactic plans for brain tumors with two different multileaf collimating systems

**DOI:** 10.1120/jacmp.v15i1.4100

**Published:** 2014-01-06

**Authors:** Livia Marrazzo, Margherita Zani, Stefania Pallotta, Daniela Greto, Silvia Scoccianti, Cinzia Talamonti, Giampaolo Biti, Marta Bucciolini

**Affiliations:** ^1^ Medical Physics Unit Careggi University Hospital Florence; ^2^ Department of Biomedical Experimental and Clinical Sciences, University of Florence Florence; ^3^ Radiotherapy Unit Careggi University Hospital Florence Italy

**Keywords:** central nervous system tumors, stereotactic radiosurgery, multileaf collimators, conformity index, homogeneity index

## Abstract

Linac‐based stereotactic radiosurgery (SRS) has been widely used for treating small intracranial lesions. This technique allows conforming the dose distribution to the planning target volume (PTV), providing a steep dose gradient with the surrounding normal tissues. This is realized through dedicated collimation systems. The present study aims to compare SRS plans with two collimating systems: the beam modulator (BM) of the Elekta Synergy linac and the DirexGroup micromultileaf collimator (μMLC). Seventeen patients (25 PTVs) were planned both with BM and μMLC (mounted on an Elekta Precise linac) using the Odyssey (PerMedics) treatment planning system (TPS). Plans were compared in terms of dose‐volume histograms (DVH), minimum dose to the PTV, conformity index (CI), and homogeneity index (HI), as defined by the TPS, and doses to relevant organs at risk (OAR). The mean difference between the μMLC and the BM plans in minimum PTV dose was 5.7%±4.2% in favor of the μMLC plans. No statistically significant difference was found between the distributions of the CI values for the two planning modalities (p=0.54), while the difference between the distributions of the HI values was statistically significant (p=0.018). For both BM and μMLC plans, no differences were observed in CI and HI, depending on lesion size and shape. The PTV homogeneity achieved by BM plans was 15.1%±6.8% compared to 10.4%±6.6% with μMLC. Higher maximum and mean doses to OAR were observed in the BM plans; however, for both plans, dose constraints were respected. The comparison between the two collimating systems showed no substantial differences in terms of PTV coverage or OAR sparing. The improvements obtained by using μMLC are relatively small, and both systems turned out to be adequate for SRS treatments.

PACS numbers: 87.53.Ly, 87.55.dk, 87.56.nk

## I. INTRODUCTION

Linac‐based stereotactic radiosurgery (SRS) is extensively used for treating small intracranial lesions. One of the main advantages exploited by this technique is the ability to conform the dose distribution to the planning target volume (PTV), providing a steep dose gradient between the PTV and the surrounding normal tissues. This is realized through the use of dedicated collimation systems with small size leaves. Commercially available micromultileaf collimators (μMLC) with narrow leaf widths are generally “add‐on” devices for use on nondedicated linac units.

Linacs with thin MLC leaf widths (4 to 5 mm at isocenter) are now routinely available. The possibility to achieve clinically acceptable treatment plans for SRS without the need of any additional devices, which require extra commissioning and an increase in treatment setup time, should be investigated.

Since 2001, at the Radiotherapy Unit of Florence University Hospital, the DirexGroup (Wiesbaden, Germany) μMLC, mounted on an Elekta (Stockholm, Sweden) Precise linac, has been used for SRS plans. About 200 patients have been treated for intracranial lesions using this device. Main advantages of the system are the small leaf size and the bidirectional leaf setting. On the other hand, the collimator needs to be manually fixed to the linac gantry prior to each usage, and it reduces the clearance around the patient, limiting the possible beam entrances.

In 2008 an Elekta Synergy linac with beam modulator (BM) was installed for treating small lesions and for SRS. This collimator does not need any manual fixation and optimizes the clearance around the patient.

It was shown by Bortfeld et al.^(1)^ that for a 6 MV photon beam the optimal leaf width according to basic physics is in the range of 1.5‐2 mm. Therefore an evaluation of the potential of the two MLCs is meaningful in order to assess their performances for SRS plans.

The present study aims to compare SRS plans obtained with the two available collimating systems in order to estimate their advantages and limitations. The comparison presented in this work is purely a computer‐based planning study and it does not aim to evaluate the dose distributions actually delivered by the two systems.

## II. MATERIALS AND METHODS

### A. Patients

The latest seventeen patients (for a total of 25 PTVs) treated with SRS for brain tumors at the Radiotherapy Department of the Florence University were planned both with the BM of the Elekta Synergy linac and the μMLC mounted on the Elekta Precise linac, using the treatment planning system (TPS) Odyssey (PerMedics Inc., San Bernardino, CA, version 4.4 and 4.6), a system capable of managing a collimator with bidirectional leaf banks.

Odyssey TPS uses for the three‐dimensional dose distribution computation the method of lateral scatter convolution. Dose is computed at each point within a 3D fan grid containing the volume of tissue irradiated by the beam. The 3D fan grid is constructed from a stack of divergent 2D grid planes perpendicular to the beam direction. The number of stack planes is set to 32, 64, or 128, according to whether the speed category is fast, medium, or slow, respectively. Both electron and photon beams are supported by the system.^(2)^

For the purposes of this work, a calculation grid with a voxel size of 0.65 mm was adopted. No inverse optimization was used for plans calculation.

In Table 1, the clinical sample characteristics are summarized (number of lesions per patient, lesion's shape, and anatomical location). For each patient the radiation oncologist identifies the gross tumor volume (GTV) on the CT. Clinical target volume (CTV) is obtained by a 1 mm isotropic expansion of GTV; PTV is obtained by a 1 mm isotropic expansion of CTV

The median and mean PTV volumes were 5.1 cm^3^ and 6.2 cm^3^ (range 0.62‐20.7 cm^3^), respectively. These volumes correspond to a median and mean diameter (obtained by approximating the volume to a sphere) of 2.1 cm and 2.2 cm (range 1.1‐3.4 cm), respectively.

Doses ranged from 10 Gy to 24 Gy and were delivered in a single fraction. The median number of beam entrances per treatment was 11 (range 8‐18).

All patients were immobilized for CT scanning and treatment using the HeadFIX frame (Elekta, Stockholm, Sweden) with an individualized vacuum cushion and a patient‐specific dental mold. This assures accuracy in patient repositioning of about 1 mm, as reported in the literature.^3^, ^4^

**Table 1:** Main features of the lesions of the selected sample

*Patient*	*Lesion*	*Anatomical Location*	*Shape*	*Distance from the Fixation Point (dental mold) (cm)*
1	1	Right parietal lobe	Spherical	17
2	1	Cerebellar vermis	Ovoidal	12
3	1	Right parietal lobe	Spherical	17
4	1	Left thalamus	Spherical	14
5	1	Left temporal lobe	Spherical	18
	2	Right occipital lobe	Spherical	16
	3	Right parietal lobe	Spherical	14
	4	Left parietal lobe	Spherical	18
6	1	Right parietal lobe	Spherical	14
7	1	Left cerebellar lobe	Spherical	14
8	1	Left frontal lobe	Spherical	12
	2	Right parietal lobe	Spherical	17
9	1	Right temporal lobe	Irregular shape	11
10	1	Left parietal occipital lobe	Spherical	16
11	1	Right parietal temporal lobe	Ovoidal	14
12	1	Left temporal lobe	Ovoidal	12
13	1	Right occipital lobe	Ovoidal	18
	2	Left frontal lobe	Spherical	15
14	1	Left occipital lobe	Irregular shape	19
15	1	Left occipital lobe	Spherical	15
	2	Right parietal lobe	Bilobed	17
16	1	Right temporal lobe	Ovoidal	12
	2	Left frontal lobe	Spherical	11
17	1	Right frontal lobe	Spherical	10
	2	Left cerebellar lobe	Spherical	12

Patients were imaged on a Brilliance Big Bore CT (Philips Healthcare, Andover, MA), with the following image acquisition parameters: 512×512 slice resolution (0.68 mm pixel size), slice thickness 2 mm.

Patient positioning before treatment was verified with a couple of portal images (antero‐posterior and latero‐lateral).

### B. Collimating systems characteristics

The μMLC consists of two levels of thin leaves (48 pairs), able to move along two orthogonal directions. The exclusive feature of bidirectional leaf setting allows one to shape irregular small fields with great accuracy. The 14 inner pairs of each level are made of thinner leaves. They form a fine resolution field of about 50 mm×45 mm at the isocenter. The maximum square field that can be defined at isocenter is 97 mm×108 mm. The μMLC is 540 mm in outer diameter, 135 mm in height, and 31 kg in weight, and must be manually mounted on the linac prior to each usage.^(5)^

Elekta Synergy BM consists of 40 pairs of individually controlled leaves with no backup jaws. The small size treatment head maximizes clearance around the patient and contributes to the wide variety of possible treatment approaches, including noncoplanar treatments.^(6)^ In Table 2, the two MLC system characteristics are compared.

**Table 2:** Main features of the two MLC systems

	μMLC	*BM*
Leaf settings	Two orthogonal leaf banks	One leaf bank
Leaves number	48 pairs	40 pairs
Physical leaf width (mm)	2.1 (14 inner pairs), 3.6 (10 outer pairs)	1.7
Isocenter leaf width (mm)	3.2 (14 inner pairs), 5.5 (10 outer pairs)^a^	4
	3.6 (14 inner pairs), 6.2 (10 outer pairs)^b^	
Max. field size (mm2)	97×108	210×160
Leaf thickness (mm)	37.5	75
Max. leakage between leaves (%)	5	2
Transmission (%)	0.4	1
Distance from the source (cm)	∼65 ^a^	39
	∼58 ^b^	
Linac head clearance (cm)	31.7	45

a ^a^ For the bank further away from the radiation source.

b ^b^ For the bank closer to the radiation source.

### C. Plan comparison

The same beam entrances were used for both plans. We tried to maintain as much as possible the parameters of the plans in order to better point out the different behaviors of the two collimating systems. For each beam entrance, the collimator rotation ensuring the better conformation was chosen. Margins to the target were the same for both plans, since they are fixed by our internal protocol in SRS. The plans obtained with the two collimating systems were normalized to the same dose at the isocenter. This is one of the possible approaches, sometimes proposed in literature.^(7)^ Plans were then compared by visual inspection of isodose lines, by dose‐volume histogram (DVH) analysis, and by using the PTV coverage, the homogeneity index (HI), and the conformity index (CI) as defined by the TPS Odyssey. Unlike the Radiation Therapy Oncology Group (RTOG) definitions,^(8)^ HI was defined by the TPS as the ratio of the maximum PTV dose ($D_{\text{max}}$) to the minimum PTV dose ($D_{\text{min}}$):

(1) $$HI=\frac{D_{\text{max}}}{D_{\text{min}}}$$

CI was defined as the ratio of the volume contained within the smallest isodose cloud that covers the target ($V_{\text{SI}}$) to the PTV volume (TV). Once the minimum PTV dose matches the prescription dose, these definitions coincide with the RTOG ones.

(2) $$CI=\frac{V_{SI}}{TV}$$

The PTV coverage was determined as the ratio of $D_{\text{min}}$ to the prescribed dose (PD).

(3) $$PT\text{V cov}erage=\frac{D_{\text{min}}}{PD}$$

PTV coverage homogeneity was evaluated by calculating the relative difference:

(4) $$PT\text{V cov}erag\text{e hom}ogeneity=\frac{\left(D_{\text{max}}-D_{\text{min}}\right)}{PD}$$

It should be noted that this parameter actually represents an estimate of the inhomogeneity of target coverage, since the higher the value of PTV coverage homogeneity, the less homogeneous the coverage.

The minimum doses have been obtained as the lowest doses to the PTV. We decided to not consider an eventual single voxel, at the PTV edge, outside the highest isodose curve encompassing the PTV. These protruding PTV voxels would affect the minimum PTV dose with modest clinical significance. Maximum doses are assessed as the ones received by the 1% of PTV volume.

Analysis was also conducted splitting PTVs into two classes according to size (“small volumes” were considered the ones $\leq 6\;{\text{cm}}^{3}$; “big volumes” the ones $>6\;{\text{cm}}^{3}$, with 6 cm^3^ being the average PTV size in our sample).

Organs at risk (OAR) were compared in terms of maximum and mean dose.

## III. RESULTS

### A. Analysis on PTVs

When looking at the PTV coverage, plans obtained with μMLC achieved better results. The minimum PTV dose was 91.3%±6.5% (± 1 SD) (range 80.1%‐98.6%) and 95.2%±6.6% (± 1 SD) (range 78.9%‐99.8%) for the BM and μMLC plans, respectively. The average difference between the μMLC and the BM plans in minimum PTV dose was 5.7%±4.2% in favor of the μMLC plans.

**Figure 1:**
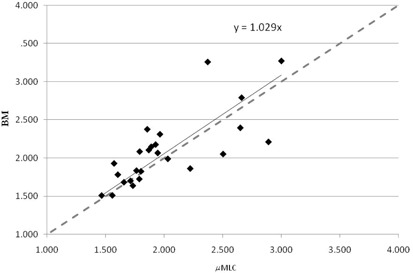
Conformity index of the BM plans versus the μMLC plans. The dashed line is the quadrant bisector.

Concerning the CI, the average value was 2.1±0.5 (± 1 SD) (range 1.5–3.3) and 2.0±0.4 (± 1 SD) (range 1.5–3.0) for the BM and μMLC plans, respectively. No statistically significant difference was found between the distributions of the CI values for the two planning modalities (p=0.54). In Fig. 1, the values obtained with BM plans are plotted versus the values obtained with μMLC. The linear fit to the experimental data is quite near to the quadrant bisector, indicating only a slight difference.

**Figure 2:**
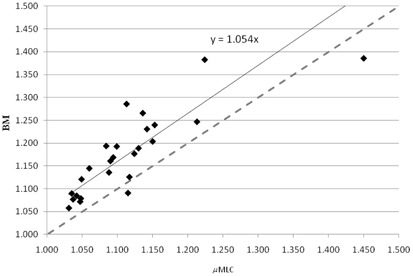
Homogeneity index of the BM plans versus the μMLC plans. The dashed line is the quadrant bisector.

Concerning the HI, the average value was 1.2±0.1 (± 1 SD) (range 1.1‐1.4) and 1.1±0.1 (± 1 SD) (range 1.0‐1.4) for the BM and μMLC plans, respectively. The difference between the distributions of the HI values for the two planning modalities was statistically significant (p=0.018). In Fig. 2, the values obtained with BM plans are plotted versus the values obtained with μMLC. In this case, the linear fit to the experimental data is quite far from the quadrant bisector, in favor of the μMLC plans.

PTV coverage homogeneity was 15.1%±6.8% for BM compared to 10.4%±6.6% of μMLC, showing a more homogeneous dose distribution for μMLC plans. Results are summarized in Table 3.

For both BM and μMLC plans, no differences were observed in CI and HI, depending on lesion size (data not shown). In the same way, no differences were observed depending on lesion shape (spherical versus differently shaped PTVs).

**Table 3:** PTVs analysis results

	μMLC	*BM*
PTV coverage (%) (± 1 SD)	95.2±6.6	91.3±6.5
CI (± 1 SD)	2.0±0.4	2.1±0.5
HI (± 1 SD)	1.1±0.1	1.2±0.1
PTV coverage homogeneity (%) (± 1 SD)	10.4±6.6	15.1±6.8

### B. organs at risk

**Figure 3:**
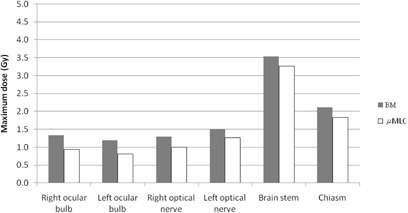
Maximum doses for selected OAR for BM and μMLC plans.

**Figure 4:**
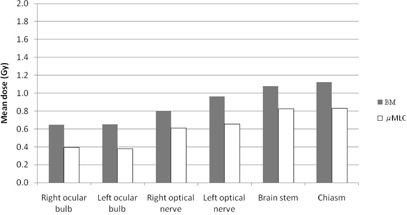
Mean doses for selected OAR for BM and μMLC plans.

In Figs. 3 and 4, maximum and mean doses to main OAR are reported for the two plan modalities. OAR doses were always slightly higher with BM plans compared to μMLC plans. However, for both plans, all the OAR doses were below their respective maximum clinical doses.

## IV. DISCUSSION

This paper deals with the comparison between SRS plans obtained with two different collimating systems and may be of interest to those acquiring dedicated MLC equipment for SRS or owning a small‐width MLC linac.

Several papers in the literature approached the issue of comparing different collimating systems for SRS. It has been found that a linac‐based SRS with multiple static beams allows better normal tissue sparing for irregularly shaped targets than multiple arcing beams using circular collimators.^9^, ^10^, ^11^ Lead alloy blocks provide an accurate conformation to the target, but are unpractical and need to be customized. The introduction of the MLC has simplified the radiotherapy process, although the target conformity can be reduced by the leaves size.

Adams et al.^(12)^ compared SRS treatment plans using a 10 mm leaf width MLC and blocks and found that, for a similar PTV coverage, a significantly higher volume of normal tissue was irradiated to >50% and >80% dose using the MLC.

Kubo et al.^(13)^ undertook a study to analyze the impact of collimator leaf width on SRS using 1.7 or 3.0 mm leaf μMLCs. They found, by analyzing CI, isodose distributions, and dose‐volume histograms, that the use of both μMLCs allows one to meet the RTOG guidelines for stereotactic radiosurgery.

Monk et al.^(14)^ dosimetrically compared the BrainLAB m3 μMLC (minimum leaf width of 3 mm) (BrainLAB, Feldkirchen, Germany) with the Varian 2100EX 5 mm leaf MLC (Varian Medical Systems, Palo Alto, CA) for SRS treatment of intracranial lesions. They found an improved CI for the 3 mm leaf width (m3) (1.5±0.2 vs. 1.6±0.2). Concerning the surrounding tissue sparing, when expressed as the maximum dose received by the adjacent critical normal tissue, there was no statistically significant difference. When expressed as the mean difference in the volume of critical structure encompassed by the 50% and 70% isodose levels, the values were 5.7% and 4.9% favoring the m3. The authors conclude that the clinical importance of these small differences is difficult to assess and individual centers may question their choice of equipment when outfitting a SRS service.

Similar conclusions were found by Tanyi et al.^(15)^ who analyzed the impact of two MLC systems (2.5 mm vs. 5 mm leaves) for linear accelerator‐based intracranial SRS. They demonstrated on 68 lesions a small dosimetric advantage of the 2.5 mm leaf width MLC system in terms of dose conformation median reduction, of normal tissue exposure, and steepness of the dose falloff. The authors pointed out that these improved dosimetric results may not translate into clinical benefits.

In the paper by Dhabaan et al.,^(16)^ the performance of a high‐definition multileaf collimator of 2.5 mm leaf width (MLC2.5) was compared to standard 5 mm leaf width MLC (MLC5) for the treatment of intracranial lesions using dynamic conformal arcs technique. The MLC2.5 shows a dosimetric advantage over the MLC5, both in treatment conformity and normal tissue sparing. The advantages are more evident when target shape complexity increases.

Nill et al.^(17)^ proposed a planning study to analyze the impact of different leaf widths on the achievable dose distributions with intensity‐modulated radiation therapy. They found the MLC with the smallest leaf width always yields the best PTV coverage. Reducing the leaf width from 4 to 2.75 mm results in a slight enhancement of the PTV coverage but in no significant improvement for most OARs. The disadvantage of the reduction of the leaf width is the increasing number of segments due to the more complex fluence patterns and, therefore, increased delivery time.

In a recent study on a new add‐on device (2.5 mm leaves), Godwin and colleagues^(18)^ investigated a range of mechanical and dosimetric characteristics which included inter‐and intraleaf leakage, light/radiation field congruence, leaf position reproducibility, radiation penumbra, total scatter factors, and mechanical rotational stability.

Asnaashari et al.^(19)^ compared the dosimetric parameters of two multileaf collimator systems, namely the BM and Radionics micro‐MLC (Radionics Inc., Burlington, MA), using measurements and Monte Carlo simulations.

In our analysis, planning outcome is evaluated in terms of PTV coverage and dose to the OAR. Concerning PTV coverage, when looking at the CI, no significant differences were observed between the two collimating systems. μMLC plans presented some advantages in terms of HI and minimum PTV dose. However, differences are relatively small and the planning outcomes with respect to PTV between the two systems are comparable. There is no obvious clinical advantage in using the μMLC compared to the BM collimator. No dependence on tumor size and shape was observed. It must be taken into account that our sample is mainly constituted of regularly shaped targets.

When looking at the OAR, slight higher maximum and mean doses are observed in the BM plans compared to the μMLC plans. This can be partly related to the higher transmission under the BM leaves due to the absence of backup jaws. Again the clinical significance of these data is debatable, since for both plans maximum OAR doses were acceptable and below the clinical threshold limit.

**Figure 5:**
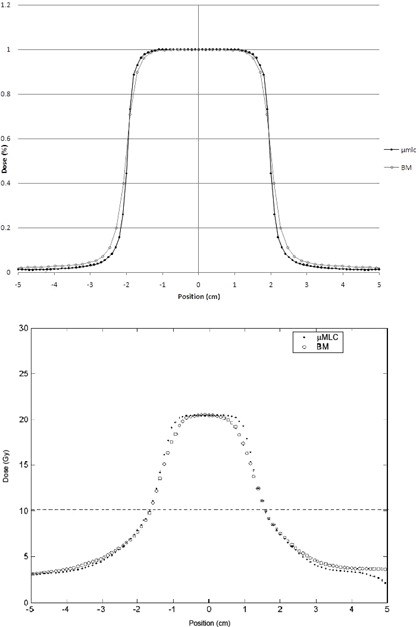
Comparison of measured profiles for (a) a μMLC and a BM 6 MV, $4\times 4\;{\text{cm}}^{2}$ field. Comparison of profiles extracted from a TPS map for (b) a μMLC plan and a BM plan. The horizontal line marks the half maximum dose.

The measured 80%‐20% penumbra $\left(P_{20-80}\right)$ for the two MLCs is summed in Table 4. Evaluation of the beam penumbra demonstrated a larger $P_{20-80}$ by up to about 1.5 mm for BM compared with the μMLC. These differences are mainly related to the different features of the two collimators leaf tip ends and their distances to the source, and they will probably have less impact on a three‐dimensional dose distribution for a multiple beam arrangement when the contribution from all beams is considered. Nonetheless, looking at the measured profiles for a $4\times 4\;{\text{cm}}^{2}$ field (Fig. 5(a)) and to those extracted by a 2D dose map for both plans (Fig. 5(b)), a difference can be observed, being the μMLC profile is flatter and characterized by a smaller penumbra, compared to BM.

**Table 4:** $P_{20-80}$ for the two MLCs

	*Field Size (cm)*	*Depth (cm)*	$P_{20-80}$ (mm)
μMLC	2.4	1.5	2.8
	8	1.5	3.2
	2.4	10	3.3
	8	10	4.6
BM	2.4	1.5	3.9
	8	1.5	4.4
	2.4	10	4.1
	8	10	6.3

## V. CONCLUSIONS

In a group of 25 lesions treated with SRS, the comparison between the two collimating systems μMLC and BM showed no substantial differences in terms of PTV coverage or OAR sparing. In particular, no significant differences were observed between the two collimating systems in terms of CI, while μMLC plans presented some advantages in terms of HI and minimum PTV dose, even though both BM and μMLC plans provided acceptable HI.

Higher maximum and mean doses to OAR were observed in the BM plans compared to the μMLC plans but, for both plans, maximum OAR doses were acceptable and below the recommended constraints. The targets we examined were mainly spherically shaped, so that these findings cannot be generalized to irregular lesions. In these conditions, when viewed quantitatively, the improvements obtained by using μMLC are relatively small and both systems turned out to be adequate for SRS treatments.

## ACKNOWLEDGMENTS

Authors wish to acknowledge Dr. Nicolò Bechini for the help in data collection.
